# Suspected Immune-Related Adverse Events With an Anti-PD-1 Inhibitor in Otherwise Healthy People With HIV

**DOI:** 10.1097/QAI.0000000000002716

**Published:** 2021-05-01

**Authors:** Cynthia L. Gay, Ronald J. Bosch, Ashley McKhann, Kendall F. Moseley, Chanelle L. Wimbish, Steven M. Hendrickx, Michael Messer, Maureen Furlong, Danielle M. Campbell, Cheryl Jennings, Constance Benson, Edgar T. Overton, Bernard J. C. Macatangay, Daniel R. Kuritzkes, Elizabeth Miller, Randall Tressler, Joseph J. Eron, William David Hardy

**Affiliations:** a Division of Infectious Diseases, the University of North Carolina at Chapel Hill, Chapel Hill, NC; b Department of Biostatistics, Center for Biostatistics and AIDS Research,Harvard T. H. Chan School of Public Health, Boston, MA; c Division of Endocrinology, Diabetes and Metabolism, Johns Hopkins University School of Medicine, Baltimore, MD; d Department of Clinical Research, Social and Scientific Systems, Inc, a DLH Company, Silver Spring, MD; e Division of Infectious Diseases, University of California, San Diego, CA; f Division of Infectious Diseases, University of Alabama at Birmingham, Birmingham, AL; g Semel Institute for Neuroscience and Human Behavior, Center for AIDS Research and Education (CARE), University of California, Los Angeles, CA; h Division of Infectious Diseases, Northwestern University, Chicago, IL; i Division of Infectious Diseases, University of Pittsburgh, Pittsburgh, PA; j Division of Infectious Diseases, Brigham and Women's Hospital, Harvard Medical School, Boston, MA; k Clinical Sciences, Global Development, Regeneron Pharmaceuticals, Inc, Tarrytown, NY; l HIV Research Branch, Division of AIDS, National Institute of AIDS, National Institutes of Health, Rockville, MD; m Division of Infectious Diseases, Johns Hopkins University School of Medicine, Baltimore, MD

***To the Editors:***

Reversing T-cell exhaustion using antibodies to immune checkpoint inhibitors (ICIs) has revolutionized cancer therapy. Because T-cell exhaustion, mediated by programmed death-1/programmed death ligand-1 (PD-1/PD-L1), may be a barrier to HIV cure^1,2^ and CD4^+^ T cells expressing PD-1 are enriched for latent HIV,^2–5^ treatment with anti-PD-1 antibodies may provide a strategy for targeting the latent HIV reservoir. Given that elimination of latently infected cells harboring replication-competent provirus will be necessary to cure HIV infection, we initiated a phase I/IIa, double-blind, placebo-controlled, dose-escalating safety and immunotherapeutic study of 2 infusions of an anti-PD-1 antibody (cemiplimab) in virally suppressed persons with HIV (PWH). Although participants were not anticipated to receive direct benefit from this study and ICIs are associated with potentially irreversible immune-related adverse events (irAEs), feedback from PWH and the scientific and HIV communities encouraged the team to pursue this HIV cure intervention. Moreover, preliminary data^6,7^ provided a reasonable expectation that cemiplimab would improve HIV-specific immune responses, reverse HIV latency, and, thus, advance the field. This risk versus benefit assessment^8^ led to the incorporation of strict measures to limit risk to participants (eg, history of autoimmune disease was exclusionary). Four of the 5 participants enrolled were randomized to receive 0.3 mg/kg of cemiplimab at weeks 0 and 6; one participant received placebo. Possible irAEs occurred in 2 participants:

## CASE 1

A 50-year-old man enrolled with baseline CD4^+^ T-cell count of 1.957 × 10^9^/L and normal thyroid-stimulating hormone (TSH) and free thyroxine (free T4) levels. Four weeks after the first infusion of cemiplimab (0.3 mg/kg), TSH of 0.02 µg/mL and free T4 of 2.73 ng/dL were consistent with hyperthyroidism (Table 1). Mild fatigue was the only symptom reported. Repeat laboratory tests at week 5 and consultation with an endocrinologist confirmed thyroiditis (Table 1), assessed as probably related to cemiplimab. Both TSH and free T4 normalized by week 24 without medical intervention. Fatigue resolved, and no new symptoms were reported.

**TABLE 1.** Laboratory Values for Participants With Possible Immune-Related AEs After Receipt of 1 Dose of 0.3 mg/kg of Cemiplimab

Case 1												
Study Week	Screen	Preentry	Entry	Wk 4	Wk 5	Wk 6	Wk 12	Wk 16	Wk 24	Wk 36	Wk 38	Wk 48
Study day	−70	−43	0	29	36	42	92	120	176	253	267	337
Thyroid laboratory test (reference range)												
TSH (0.27–4.2 mIU/L)	1.91	3.91		**0.02**	**<0.01**	**0.01**	3.97	2.87	1.55	**6.67**	1.55	3.5
Thyroxine (4.5–10.9 mcg/dL)		7.1		**13.4**	**16.7**							
Free T4 (0.93–1.7 ng/dL)				**2.73**	**4.1**	**3.06**	**0.81**	**0.86**	1.01	1.20	1.12	1.02
Thyroglobulin antibody (0.0–4.0)					**80.4**							
TSH receptor antibody (≤122)					<0.09							

Case 2																
Study Week	Screen	Preentry	Entry	Wk 2	Wk 2.1	Wk 2.2	Wk 4	Wk 5	Wk 6	Wk 12	Wk 16	Wk 20	Wk 24	Wk 28	Wk 36	Wk 48
Study day	−64	−48	0	14	17	21	28	35	42	84	116	140	168	204	253	337
Liver laboratory test (reference range)																
AST (0–40 IU/L)	30	23	**53**	**261 (G3)**	**192 (G2)**	**149 (G2)**	**61**	**50**	39	27	23	**82**	35	**62**	**54**	**54**
ALT (0–44 IU/L)	23	21	**58**	**287 (G3)**	**272 (G2)**	**238 (G2)**	**93 (G1)**	**48**	31	19	20	**66**	41	**65**	**49**	**56**
Total bilirubin (0.0–1.2 mg/dL)	0.8	0.3	0.3	0.4	1	0.8	0.5	0.6	0.4	0.2	0.4	0.3	0.4	0.4	0.5	0.7
Prothrombin time (10.2–12.8 s)						11.1	10.7	11								

(Bold Type = Outside Reference Range)

G, grade.

## CASE 2

A 57-year-old man with baseline CD4^+^ T-cell count of 0.911 × 10^9^/L had normal aspartate aminotransferase (AST) and alanine aminotransferase (ALT) levels at screening. Just before the first infusion of cemiplimab (0.3 mg/kg), asymptomatic grade 1 elevations in AST and ALT levels were observed (Table 1). Routine safety assessment 2 weeks after the first infusion revealed asymptomatic grade 3 elevations in AST and ALT levels (Table 1). On further questioning, the participant reported acetaminophen (500 mg × 1) and alcohol use (6 beer and 2 whiskey drinks) the evening before the week 2 visit. Hepatology consultation revealed no autoimmune etiology or hepatic synthetic dysfunction but elicited chronic alcohol use. The pattern of the hepatic enzyme elevations and their slow resolution were deemed inconsistent with acute alcohol toxicity and, therefore, judged to be possibly related to cemiplimab. Elevated AST and ALT levels resolved 35 days postinfusion without intervention. Liver biopsy was not pursued, given the participant's asymptomatic course and gradual improvement without intervention. This significantly limited definitive assessment of causality due to drug-induced liver injury versus immune-related hepatitis versus the contribution of acute or chronic alcohol use.

Per protocol-specified management of suspected irAEs, the second infusion at week 6 was held for both participants. A detailed, unblinded review of safety data from both cases by the independent Safety Monitoring Committee (SMC) was triggered and all study infusions held. Because of the probability of one irAE and the possibility of a second irAE, the SMC recommended halting accrual of additional study participants and holding further cemiplimab infusions. Of note, 2 participants who received 2 cemiplimab infusions before the occurrence of these events remained asymptomatic without laboratory abnormalities. All 4 cemiplimab-treated participants completed the study with no further irAEs or other safety events through 48 weeks after first cemiplimab infusion.

The irAEs similar to these 2 cases are well described with other ICIs and frequently managed in cancer patients receiving this immunotherapy,^9,10^ although the resolution of the participant's thyroid abnormality in this study has not been commonly described. The irAEs can occur after a single infusion, although typically associated with higher doses, and as early as 14 days postinfusion. Given the lack of anticipated direct benefit to study participants and the frequency of possible/probable irAEs (2 of 4 participants) at the lowest dose of study drug, the study was closed to accrual. Of note, ICIs have shown an acceptable risk–benefit profile in PWH treated for cancer in previous studies.^11^ Whether well-suppressed HIV infection in otherwise healthy individuals without cancer contributed to risk of irAEs in this study remains unknown.

The reduction or elimination of latent HIV reservoirs in PWH receiving suppressive antiretroviral therapy will likely require a combination of multiple therapeutic modalities including interventions that enhance HIV-1–specific immune responses to clear or contain these cells when activated to express replication-competent virus. Strategies to reverse HIV-specific immune exhaustion and target latently infected cells must be tested. These may require more targeted PD-1 blockade than that obtained with systemic administration of antibodies, coupled with a better understanding of risks for immune-mediated adverse events, to pursue studies of ICIs in otherwise healthy, virologically suppressed PWH. Our experience underscores the value of the multiple, carefully considered steps to minimize risk to study participants built into this study. These included engagement with representatives of the PWH community before, during, and after the study, highly restrictive entry criteria, active participation of physician investigators in the informed consent process, written assessment of understanding to document the adequacy of the informed consent process, frequent safety visits and phone contact with participants after each infusion, a 6-week observation period between infusions for safety assessment, and a detailed, predetermined toxicity management plan incorporating rapid review by our SMC in response to suspected irAEs. This study underscores the potential challenges of translating successful immunotherapeutic interventions from the high morbidity/mortality cancer field to otherwise healthy virologically suppressed PWH.
